# Test performance data demonstrates utility of a cattle DIVA skin test reagent (DST-F) compatible with BCG vaccination

**DOI:** 10.1038/s41598-022-16092-8

**Published:** 2022-07-14

**Authors:** Gareth J. Jones, Timm Konold, Shellene Hurley, Tom Holder, Sabine Steinbach, Mick Coad, D. Neil Wedlock, Bryce M. Buddle, Mahavir Singh, H. Martin Vordermeier

**Affiliations:** 1 TB Immunology and Vaccinology, Department of Bacteriology, Animal and Plant Health Agency, New Haw, Addlestone, KT15 3NB Surrey UK; 2 AgResearch, Palmerston North, New Zealand; 3 Lionex Diagnostics and Therapeutics GmbH, Braunschweig, Germany

**Keywords:** Tuberculosis, Vaccines

## Abstract

Bacillus Calmette-Guérin (BCG), an attenuated strain of *Mycobacterium bovis (M. bovis)*, is the lead candidate vaccine for control of bovine tuberculosis (TB) in cattle. However, BCG vaccination sensitises cattle to bovine tuberculin, thus compromising the use of the current bovine TB surveillance tests. To address this, we have developed a diagnostic skin test that is not compromised by BCG vaccination and is able to detect BCG vaccinated animals that subsequently develop bovine TB following exposure to *M. bovis*. Building on previous work using ‘in house’ formulated protein cocktail reagents, we herein present test performance data for a single fusion protein (DST-F) containing the mycobacterial antigens ESAT-6, CFP-10 and Rv3615c formulated as a ‘ready to use’ reagent by a commercial manufacturer. Our results demonstrate that, unlike tuberculin reagents, a diagnostic skin test using DST-F maintained high specificity in BCG vaccinated animals. Furthermore, the DST-F skin test demonstrated a high relative sensitivity in identifying *M. bovis* infected animals, including those where BCG vaccination failed to prevent bovine TB pathology following experimental exposure to *M. bovis*. The DST-F is currently undergoing field trials in Great Britain to support its licensure and commercialisation.

## Introduction

Bovine tuberculosis (TB) is a disease that affects important livestock species across the world. Although the disease is caused by pathogens of the *Mycobacterium tuberculosis* group of mycobacteria, in many countries (including the United Kingdom) bovine TB is caused almost exclusively by *M. bovis*. Bovine TB is an important source of economic loss through both reduced productivity and the cost of control programmes, with a global economic loss estimated at around US$3 billion per year^1^. Furthermore, bovine TB has important zoonotic consequences, particularly in low to middle income countries where pasteurisation of milk from livestock cannot be guaranteed^2^.

In the UK, like in many high income countries, a test and slaughter strategy is being employed that is based on the Single Intradermal Comparative Cervical Tuberculin (SICCT) test, with an interferon-gamma release assay sometimes applied as an ancillary test to maximise the detection of infected animals. However, this control strategy comes at a high cost, both financially where it is estimated that tackling bovine TB in England alone costs the taxpayer around £70 million a year with additional costs of £50 million to farmers^3^, but also ethically where around 38,000 cattle were slaughtered in GB between July 2020 and June 2021 to tackle the disease. Therefore, development of a cattle vaccine against bovine TB is a high research priority.

To date, the only available vaccine for bovine TB is Bacillus Calmette-Guérin (BCG), an attenuated strain of *M. bovis*, and efficacy studies evaluating the effect of BCG vaccination against experimental *M. bovis* infection in cattle have demonstrated promising results^4,5^. Furthermore, the efficacy of BCG vaccination has also been demonstrated in field studies^6–9^. However, although studies demonstrate that BCG vaccination reduces the severity of bovine TB disease, they also highlight that it is not fully protective in all vaccinates^10–15^. Thus, diagnostic tests are still required to detect BCG vaccinated animals that develop bovine TB following exposure to *M. bovis*. Unfortunately, BCG vaccination sensitises cattle to the current diagnostic skin test reagents (i.e. purified protein derivatives of tuberculin) which precludes their use in this setting^16^. Therefore, to ensure the continuation of a test and slaughter-based control strategy in the setting of BCG vaccination, research efforts have focused on developing a diagnostic skin test that: (i) does not induce false positive skin test reactions in BCG vaccinated animals; and (ii) is able to detect BCG vaccinated animals that subsequently develop bovine TB following exposure to *M. bovis*. Initial development of this so called ‘DIVA skin test’ (DIVA: Detecting Infected amongst Vaccinated Animals) was based on assessing cocktails of recombinant mycobacterial proteins^17^ and resulted in prioritising the proteins ESAT-6, CFP-10 and Rv3615c^18^, whilst further refinement explored the use of these as a single fusion protein reagent formulated ‘in house’^19^. To extend these studies, we have worked closely with a commercial manufacturer to produce a ‘ready to use’ DIVA skin test fusion protein reagent (DST-F), and the performance data for this reagent are presented herein.

## Results

**Figure 1:**
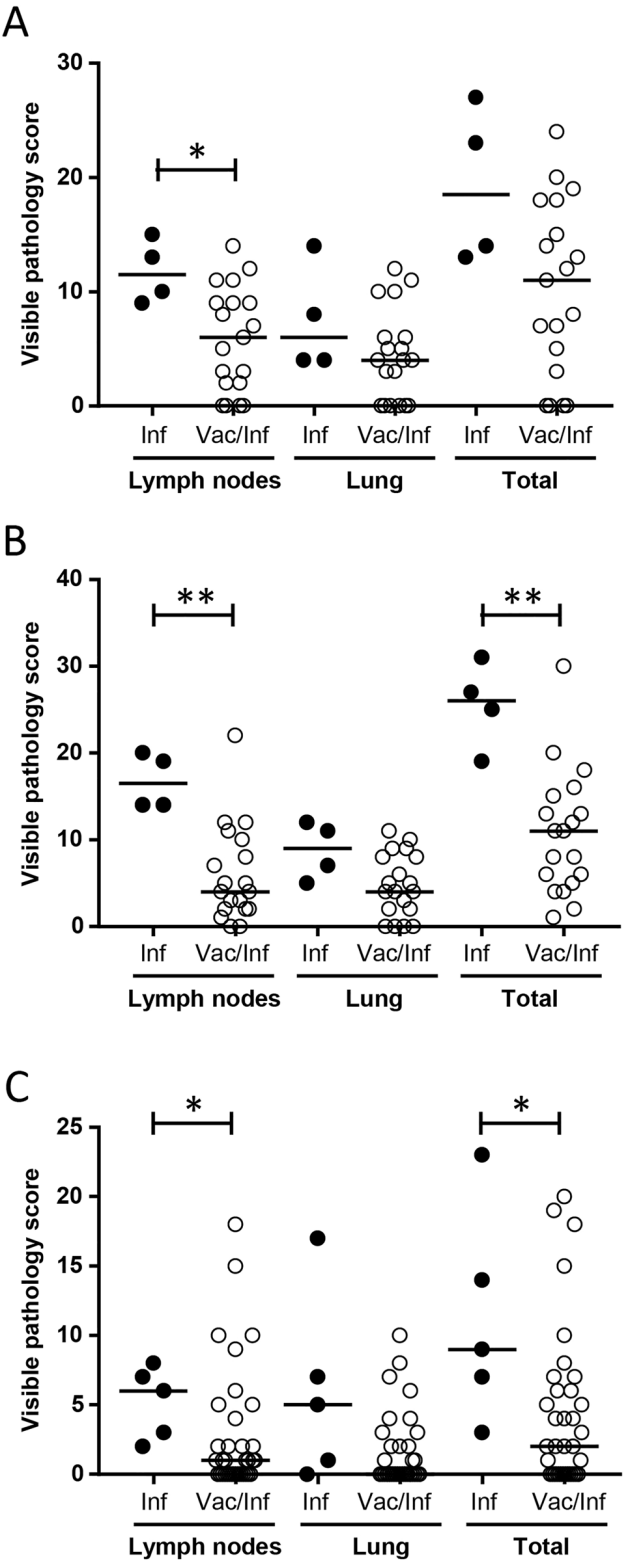
Reduction of bovine TB visible pathology in BCG vaccinates. Lymph node, lung and total pathology scores for the BCG vaccinated/*M. bovis* infected (Vac/Inf) and the non-vaccinated/*M. bovis* infected controls (Inf) from study 4 (**A**), study 5 (**B**) and study 6 (**C**). Each symbol represents an individual animal while horizontal bars represent group median values. *p < 0.05, **p < 0.01, Mann Whitney Test.

In order to assess the ability of the DST-F to identify infected cattle, a series of experimental *M. bovis* infection studies (Table 1; studies 1–3) were conducted. Post mortem examination for bovine TB pathology and culture of post mortem tissue samples confirmed *M. bovis* infection in all 71 animals studied (data not shown). In total, 68 of these animals tested positive to the DST-F (Table 2), which was greater than that seen for the SICCT test (62 test positives) and only slightly less than that seen for the SIT test (70 test positives). Although this provided encouraging performance data for the DST-F in *M. bovis* infected animals, the true target group of animals for this test are BCG vaccinated cattle that fail to be fully protected from development of bovine TB. Thus, in order to assess the ability of the DST-F to identify BCG vaccinated cattle that showed signs of bovine TB following experimental exposure to *M. bovis*, we performed three separate vaccine/challenge experiments (studies 4–6). Again, at the end of each experiment, all animals were subjected to a detailed post mortem examination where signs of bovine TB pathology were scored and samples of lymph nodes and lung tissue collected for *M. bovis* culture. The results of these investigations are summarised in Fig. 1. In all three experiments, the level of visible pathology in lymph node tissue was significantly lower in the vaccinated/infected group of animals compared to the infected only control group in that experiment. Similarly, in two of the three experiments the total visible lesion score was also significantly lower in this group of animals. Thus, all three experiments demonstrated a protective effect of BCG vaccination on the severity of bovine TB pathology following experimental infection with *M. bovis*. However, these results also indicated that although BCG limits the severity of bovine TB pathology, it did not prevent *M. bovis* infection per se, as *M. bovis* could be cultured from post mortem tissue from all but one of the 73 BCG vaccinated/*M. bovis* infected animals (data not shown). Thus, we assessed the ability of the DST-F reagent to detect the 72 culture confirmed BCG vaccinated/*M. bovis* infected animals. Of these 72 animals, 65 tested positive to the DST-F reagent (Table 2), which was greater than that seen for the SICCT test (61 test positives) but less than that seen for the SIT test (72 test positives). For the non-vaccinated/*M. bovis* infected control animals from these studies, all showed evidence of *M. bovis* infection and, with the exception of one animal in study 6, tested positive to the DST-F reagent. All animals in these groups also tested positive to the SICCT and SIT tests.

**Table 1:** Overview of the cattle studies for DST-F performance data.

Study	Number of animals	Age	Breeds (number)	Sex (number)	BCG dose (CFU)	*M. bovis* infection		Skin test
						Week post vaccination	Dose (CFU)	
1	23 infected only	6 to 8 months	F (22), H (1)	Male (23)	N.A	N.A	4.0 × 10^3^	6 weeks post infection
2	24 infected only	6 to 8 months	F (21), Ayr (2), HF (1)	Male (24)	N.A	N.A	36.0 × 10^3^	5 weeks post infection
3	24 infected only	8 to 12 months	HF (24)	Male (24)	N.A	N.A	8.0 × 10^3^	5 weeks post infection
4	19 vaccinated/infected 4 infected only	6 to 8 months	HF (23)	Male (23)	0.2 × 10^6^	10	5.6 × 10^3^	14 weeks post infection
5	19 vaccinated/Infected 4 infected only	4 to 6 months	HF (14), BF (7), H (2)	Male (23)	1.0 × 10^6^	8	5.2 × 10^3^	12 weeks post infection
6	35 vaccinated / infected 5 infected only	12 months	FxH (40)	Female (40)	1.0 × 10^6^	9	1.6 to 2.6 × 10^5^	8 weeks post infection
7	25 non-infected controls	8 to 12 months	HF (20), BF (5)	Male (25)	N.A	N.A	N.A	N.A
8	20 non-infected controls	40 to 54 days	H (20)	Male (16) Female (4)	N.A	N.A	N.A	N.A
9	20 BCG vaccinated	43 to 57 days	H (20)	Male (16) Female (4)	1 to 4 × 10^6^ (N.T.)	N.A	N.A	7 weeks post vaccination

HF, Holstein–Friesian; H, Holstein; BF, British Friesian; FxH, Friesian cross Hereford; F, Friesian; Ayr, Ayrshire;

N.A., Not applicable; N.T., Not titrated.

**Table 2:** Skin test performance data for the DST-F reagent.

Study	Animal group	Skin test results (n/N)		
		DST-F^a^	SICCT^b^	SIT^c^
1	Infected	21/23	14/23	22/23
2	Infected	24/24	24/24	24/24
3	Infected	23/24	24/24	24/24
4	Infected	4/4	4/4	4/4
	Vaccinated/infected	17/19	18/19	19/19
5	Infected	4 / 4	4/4	4/4
	Vaccinated/infected	19/19	18/19	19/19
6	Infected	4/5	5/5	5/5
	Vaccinated/infected	29/34	25/34	34/34
7	Non-infected controls	0/25	0/25	0/25
8	Non-infected controls	0/20	0/10^d^	0/10^d^
9	BCG vaccinated	0/20	7/10^d^	10/10^d^

*n*, number positive; *N*, total number tested.

^a^Response considered positive if Δ skin thickness for DST-F ≥ 2 mm.

^b^Response considered positive if Δ skin thickness for PPDB—PPDA > 4 mm (standard OIE and UK interpretation).

^c^Response considered positive if Δ skin thickness for PPDB ≥ 4 mm.

^d^Tuberculin reagents were tested in only a subset of animals.

In order to assess the level of false-positive results induced by the DST-F, skin tests were performed in three groups of non-infected animals (studies 7 to 9) and the results are summarised in Table 2. In the two studies with non-infected control animals, no false positive responses to either the DST-F or the tuberculin reagents were observed. Similarly, no false-positive responses to the DST-F were observed in the 20 BCG vaccinated calves (study 9). In contrast, a high proportion of the BCG vaccinated calves that were tested with tuberculin reagents gave false-positive responses, with 7/10 and 10/10 testing positive to the SICCT and SIT test respectively.

The skin test data generated from these nine studies were combined and are summarised in Table 3. The relative sensitivity of the DST-F at detecting *M. bovis* infected animals was 95%, which was higher than that for the SICCT test (89%) but lower than that for the SIT test (99%). However, none of these differences achieved statistical significance. The relative sensitivity of the DST-F in detecting BCG vaccinated animals that showed signs of bovine TB following experimental *M. bovis* infection was slightly lower at 90%. Again, this was higher than that for the SICCT test (85%), but significantly (*p* < 0.05, McNemar’s test) lower than that for the SIT test (100%). None of the three skin tests induced false-positive responses in non-infected control animals, resulting in specificity estimates of 100%. Similarly, no false-positive responses to the DST-F were observed in BCG vaccinated animals, again resulting in a specificity estimate of 100%. In contrast, significantly lower values for the SICCT (*p* < 0.05, McNemar’s test) and SIT (*p* < 0.01, McNemar’s test) tests were observed in this group of animals, with specificity estimates of 30% and 0% respectively.

**Table 3:** Relative sensitivity and specificity estimates for DST-F.

Animal group	Test performance	DST-F^a^	SICCT^b^	SIT^c^
*M. bovis* infected	% Sensitivity [95% CI] (n/N)^d^	95 [88, 98] (80/84)	89 [81, 94] (75/84)	99 [94, 100] (83 / 84)
BCG vaccinated / *M. bovis* infected	% Sensitivity [95% CI] (n/N)	90 [81, 95]^e^ (65/72)	85 [75, 91] (61/72)	100 [95, 100] (72/72)
Non-infected controls	% Specificity [95% CI] (n/N)	100 [92, 100] (0/45)	100 [90, 100] (0/35)	100 [90, 100] (0/35)
BCG vaccinated	% Specificity [95% CI] (n/N)	100 [84, 100] (0/20)	30 [11, 60]^f^ (7/10)	0 [0, 28]^g^ (10/10)

^a^Response considered positive if Δ skin thickness for DST-F ≥ 2 mm.

^b^Response considered positive if Δ skin thickness for PPDB—PPDA > 4 mm (standard OIE and UK interpretation).

^c^Response considered positive if Δ skin thickness for PPDB ≥ 4 mm.

^d^*n*, number positive; *N*, total number tested.

^e^*p* < 0.05 (compared to SIT), McNemar’s test.

^f^*p* < 0.05 (compared to DST-F), McNemar’s test.

^g^*p* < 0.01 (compared to DST-F), McNemar’s test.

## Discussion

Our initial development of a DIVA skin test reagent was based on cocktails of recombinant mycobacterial proteins that were immunogenic in *M. bovis* infected cattle but did not induce responses in naïve or BCG vaccinated animals^17^, and a cocktail comprising of ESAT-6, CFP-10 and Rv3615c proteins was prioritised for evaluation in different cattle groups. The results of these analyses demonstrated a high specificity of this reagent when tested in BCG vaccinated animals or uninfected controls, whilst retaining a relative sensitivity for detection of infection similar to that of the tuberculin SICCT test when evaluated at standard interpretation^18^. However, from a manufacturing point of view, production of a single fusion protein reagent has many advantages over one consisting of a cocktail of three individual proteins. For example, only a single production run is required for the fusion protein, as opposed to three separate production runs required for the components of the cocktail. In addition, compared to producing the cocktail reagent, production of a single fusion protein would reduce the required associated quality control data by a third. Lastly, there would be no need to have a system in place to formulate a cocktail reagent from the three individually produced components. Proof of concept data demonstrated that a fusion protein of ESAT-6, CFP-10 and Rv3615c induced in vitro IFN-γ production using peripheral blood mononuclear cells from *M. bovis* infected cattle but not from naïve controls or BCG vaccinated animals^19^. Furthermore, this fusion protein generated positive skin reactions in *M. bovis* infected animals but not in naïve controls when formulated ‘in house’ as a skin test reagent^19^. In order to further develop this reagent into a potential commercial product, we worked with the manufacturer (i.e. Lionex) to produce a fusion protein DIVA skin test reagent (referred to as DST-F) that could be used ‘straight from the bottle’ in a similar manner to tuberculin reagents. Furthermore, quality control protocols and batch release criteria (see material and methods) were developed to provide assurance of batch-to-batch consistency, bearing in mind potential scale up to commercial manufacture in line with Good Manufacturing Practice standards. In total, DIVA skin test reagents based on the cocktail and fusion protein formulations have been tested ‘head-to-head’ in 191 individual animals (unpublished data). Statistical analysis assessing the test outcome of the two reagents demonstrated an observed agreement of 94% with a Cohen’s kappa coefficient of 0.885 (95% CI of 0.819 to 0.951), indicating an ‘almost perfect agreement’^20^ between the two reagents (data not shown). Thus, given the manufacturing advantages of the fusion protein, the DST-F reagent was selected as the candidate DIVA skin test reagent to take forward.

As summarised in Table 3, the relative sensitivity of the DST-F in identifying confirmed experimentally *M. bovis* infected animals was 95% (95% CI of 88% to 98%), which was greater than that for the SICCT test performed at the same time in these animals. However, it should be noted that this relative sensitivity estimate was generated using experimentally infected animals and may not fully represent DST-F test performance when used in naturally infected cattle. Indeed, previously published data for the DIVA skin test cocktail formulation demonstrated a significantly lower proportion of test positives in naturally infected animals compared to experimentally infected animals (78 and 100% respectively; *p* < 0.05, Fisher’s exact test), although it should be highlighted that the proportion of naturally infected animals testing positive to the standard SICCT test was also around 76%^18^. In addition, our initial proof of concept data for the ‘in house’ fusion protein formulation demonstrated a similar outcome, with 76% of naturally infected animals (16 out of 21) compared to 100% of experimentally infected animals (6 out of 6) giving a skin test positive result (data not shown). Thus, one focus of our current research is to assess the performance of the DST-F further in increased numbers of naturally infected cattle to provide statistically robust precision calculations of the DST-F test sensitivity in a realistic scenario to support future DST-F test validation exercises.

Numerous vaccination / challenge studies over the past 25 years demonstrate that BCG vaccination reduces the severity of bovine TB disease^4^. However, given that BCG vaccinated animals can still develop bovine TB (albeit with less severity), it is crucial that we understand the ability of diagnostic reagents to detect these animals. To this end, we performed three BCG vaccination / *M. bovis* infection experiments to assess the relative sensitivity of the DST-F in detecting animals that showed evidence of bovine TB disease after exposure to *M. bovis*. The results of these studies demonstrated a high relative sensitivity of 90% (95% CI of 81% to 95%), which did not differ significantly (*p* = 0.348, Fisher’s exact test) from the value estimated in non-vaccinated / *M. bovis* infected animals (Table 3). Compared to non-vaccinated animals, BCG vaccinated cattle showed reduced numbers of TB lesions, severity of TB lesions and numbers of viable bacteria recovered from affected tissue^11,15,21–23^. Thus, it might be expected that this reduced disease burden results in lower ongoing activation of effector immune responses to mycobacterial antigens, which in turn may compromise the sensitivity of diagnostic tests that utilise these responses as a readout. With that in mind, it is encouraging that the relative sensitivity of the DST-F remained high in the target animal group, i.e. BCG vaccinated animals that demonstrated evidence of bovine TB following exposure to *M. bovis*.

To generate specificity data, the DST-F reagent was tested in two groups of animals: (i) non-infected calves and (ii) BCG vaccinated calves. As shown in Table 3, the results of these studies were in line with previous published data for the DIVA skin test cocktail^18^ and confirmed that the DST-F reagent maintained the high level specificity required of a DIVA test compatible with BCG vaccination under experimental conditions. To provide additional reassurance of this, further assessment of DST-F specificity is currently planned in field trials (see below).

The DST-F performance data presented herein formed part of an application to the UK Veterinary Medicines Directorate for an Animal Test Certificate (ATC) to enable commencement of field trials for the DST-F. This application also contained a summary of the manufacturing process (provided by Lionex) and the results of a safety study conducted by the Animal and Plant Health Agency (APHA) to Good Laboratory Practice standards, where a single and repeat dose of DST-F were assessed in both naïve and BCG vaccinated calves by monitoring the injection site and general health parameters (including temperature, clinical observations and general behaviour). The conclusion of this study was that the intradermal administration of DST-F either alone or concurrently with administration of tuberculin skin test reagents did not cause any adverse local or systemic effects in naïve or BCG vaccinated calves (data not shown). The ATC was successfully awarded, and field trials are currently underway in the UK to initially confirm the safety and specificity of the DST-F reagent in a larger number of unvaccinated cattle in field situations, before continuing to field evaluation in a large number of BCG vaccinated cattle, with the intention that the data generated from these studies will support a future Marketing Authorisation for the DST-F reagent.

## Materials and methods

### Animals

#### *M. bovis* experimental infection experiments

A total of 3 studies were performed (Table 1; studies 1–3). In all cases, male calves (between 6 and 12 months of age) were sourced from officially bovine TB free herds in GB that had no recent history of bovine TB and were housed at APHA Weybridge throughout the experiment. Skin tests were performed around 5 to 6 weeks post infection. Animal details are summarised in Table 1.

### BCG vaccination/*M. bovis* challenge experiments

A total of 3 studies were performed (Table 1; studies 4–6). For the two experiments conducted at APHA Weybridge (study 4 and 5), male calves (between 4 and 8 months of age) were sourced from officially bovine TB free herds in GB that had no recent history of bovine TB and were housed at APHA Weybridge throughout the experiment. For the experiment conducted by AgResearch (study 6), female cattle (approximately 12 months of age) were obtained from TB free accredited herds from an area of New Zealand where farmed and feral animals were free of TB. The animals were kept and vaccinated at the AgResearch Aorangi farm, whilst immediately prior to challenge with *M. bovis* the cattle were transported to the TB containment unit in the AgResearch Kaitoke farm. Skin tests were performed between 8 and 14 weeks post infection. Animal details are summarised in Table 1.

### Non-vaccinated non-infected control calves

A total of 2 studies were performed (Table 1; studies 7 and 8). For study 7, male calves (between 8 and 12 months of age) were sourced from officially bovine TB free herds in GB that had no recent history of bovine TB and were housed at APHA Weybridge throughout the experiment. For study 8, calves (40–54 days old) were enrolled in a DST-F safety study conducted to Good Laboratory Practice (GLP) quality standards to generate data to support an Animal Test Certificate application to the Veterinary Medicines Directorate (Weybridge, UK). As there was an essential requirement from the regulator to demonstrate these calves were bovine TB free, they were sourced from a known TB free location, Denmark. Animal details are summarised in Table 1.

### BCG vaccinated calves

Similar to that described above, calves (43–57 days old) were also enrolled in a DST-F safety study in BCG vaccinated calves. Skin tests were performed 7 weeks post BCG vaccination. Animal details are summarised in Table 1 (study 9).

### Ethical approval

All experiments involving live animals were performed in accordance with relevant guidelines and regulations, and in compliance with the ARRIVE guidelines. Animal procedures and experimental protocols were approved by a named institutional committee: for work conducted at APHA this was the APHA Animal Welfare and Ethical Review Board (references 70/7737–2-007, PF7D840A5-2-004v, 70/7737–1-012 and PFFFE51F-2–002), while for work conduced at AgResearch this was the Grasslands Animal Ethics Committee (reference AE 14,741).

### BCG vaccination

BCG Danish strain 1331 (AJVaccines, Copenhagen, Denmark) was used for all vaccinations. All BCG inocula were prepared on the day of vaccination by reconstituting each vial of BCG with 1 ml of Sauton diluent and 0.5 ml was administered subcutaneously to the animals. The inoculum used was titrated by plating out serial dilutions to calculate the actual dose delivered (Table 1). In the vaccine/challenge experiments, non-vaccinated control animals received either no BCG vaccine (study 6) or a 0.5 ml subcutaneous injection of phosphate buffered saline (PBS) (studies 4 and 5).

### Experimental infection

Animals in studies 1 to 6 were experimentally infected via the endotracheal/endobronchial route with a virulent strain of *M. bovis* (AF2122/97 for studies 1 to 5; 83/6235 for study 6). Briefly, for studies 1 to 5, calves were sedated with 2% Rompun (0.12 ml/50 kg, i.v. route; reversal with the same dose of Antisedan, i.v. route) prior to insertion of an endoscope through the nasal cavity into the trachea for delivery of the inoculum (2 ml) through a 1.8 mm internal diameter cannula just below the main bifurcation between left and right lobes. Two ml of PBS were used to rinse any remains of the inoculum from the cannula, and then the cannula and endoscope were withdrawn and cannula discarded. The canal through which the cannula was inserted into the endoscope was rinsed with 20 ml of sterile water and the outside of the endoscope was wiped with sterilizing wipes prior to infection of the next animal. For study 6, calves were infected as previously described^21^. The *M. bovis* in the inoculum used to infect the cattle was cultured to determine CFUs. For the vaccine / challenge experiments (studies 4 to 6), animals were infected around 8 to 10 weeks post vaccination. Details of the challenge dose and time of challenge are summarised in Table 1.

### Post mortem examinations

Post mortem examinations (PME) were performed on the infected animals at the end of the experiment to quantify the level of TB pathology observed in the lungs and lymph nodes using a modification of a scoring system previously published^24^. In the majority of studies, the following lymph nodes were examined: left and right submandibular; left and right medial retropharyngeal; cranial mediastinal; caudal mediastinal; left and right bronchial; and cranial tracheobronchial. The exceptions were studies 4 and 5, where due to logistical and practical reasons the head lymph nodes (submandibular and medial retropharyngeal) were not examined. Each lymph node was serially sliced in 1 mm thick sections and a pathology score applied based on the number and extent of lesions observed using the following criteria: score 0 for no visible lesions; score 1 for a single small lesion (1–2 mm diameter), score 2 for two to five small lesions or a single large area (at least 5 mm by 5 mm); score 3 for between five and twenty small lesions or two to three large areas; score 4 for more than twenty small lesions or more than three large areas; and score 5 for consolidated pathology of over 50% of the lymph node. In addition, each lobe of the lungs was serial sliced in 5–10 mm sections and scored in the following way: score 0 for no visible lesions; score 1 for a single small lesion (< 10 mm diameter); score 2 for two to five small lesions; score 3 for between five and twenty small lesions or a single large area (> 10 mm diameter); score 4 for more than twenty small lesions or more than one large area; score 5 for a substantial affected area of the lung containing lesions. Samples of lymph nodes and lung tissue were also collected at post mortem for *M. bovis* culture.

### Culture of *M. bovis* from tissue samples

Tissues collected at PME were stored frozen at − 80 °C prior to processing. Briefly, individual samples were thawed, homogenised in 10 ml of PBS using a Seward Stomacher Paddle Blender and spread onto Modified 7H11 agar plates^16^. Plates were incubated at 37 °C for a minimum of 4 weeks prior to enumeration of individual colonies on the plates.

### Skin test reagents

Purified protein derivatives from *M. avium* (PPD-A; 25,000 IU/ml) and *M. bovis* (PPD-B; 30,000 IU/ml) were obtained from a commercial manufacturer (Thermo Fisher, UK). The DST-F reagent, consisting of a histidine-tagged fusion recombinant protein of ESAT-6, CFP-10 and Rv3615c in PBS was produced by a commercial manufacturer (Lionex, Braunschweig, Germany). Briefly, the DST-F fusion protein was expressed in *Escherichia coli* (*E. coli*), purified by nickel affinity chromatography, refolded against 10 mM NH_4_HCO_3_ (pH 8.0), buffer exchanged against PBS (pH 7.4) and finally further diluted in PBS to a concentration of 300 µg/ml prior to bottling in EP type 1 glass vials with closure stoppers and sealed with aluminium caps. Batch release criteria included: protein content of 300 µg/ml ± 5%; Western blot demonstration of a positive reaction when using anti-histidine tag and protein specific antibodies but no reaction with an anti-*E. coli* antibody; a purity of greater than 95% using SDS-PAGE and densitometry analysis; endotoxin content of less than 25 IU/mg; and sterility compliant with European Pharmacopoeia monograph 2.6.

### Skin test procedure

Skin testing was carried out in compliance with the Instructions for Tuberculosis Testing in Bovines, available through the APHA Vet Gateway. Briefly, injection sites located in the border of the anterior and middle third of the neck on either side of the animal were clipped and pre-injection skin thickness recorded. Tuberculin and DST-F skin tests were performed by intradermal injection of 0.1 ml of PPD-A and PPD-B, or DST-F reagent respectively. After 72 h, the skin test sites were checked for reactions by palpation of the skin. If palpable reactions were detected, the skin thickness at the injection site was re-measured and recorded. If no palpable reaction was detected, the pre-injection values were used. The exception to this were the GLP safety studies (studies 8 and 9), where the skin thickness at 72 h was measured automatically. Results are expressed as the increase in skin thickness at 72 h compared to the thickness pre-injection. The timings of the skin test for each experiment are detailed in Table 1.

### Statistical analysis

Analyses were performed using GraphPad Prism 7 (GraphPad Software, USA). The degree of pathology between BCG vaccinated and control calves was compared using the Mann–Whitney test. The McNemar matched pair test or the Fisher’s exact test were used where appropriate to compare proportions of test positives and negatives. A p value of less than 0.05 was considered statistically significant.

## Data Availability

The data sets used and/or analysed during the current study are available from the corresponding author on reasonable request.
